# Supplementary material for: Smart load balancing in cloud computing: Integrating feature selection with advanced deep learning models
Source: PLoS One. 2025 Sep 9;20(9):e0329765. doi: 10.1371/journal.pone.0329765 (PMC12419607; doi:10.1371/journal.pone.0329765)
Supplement: Attachment — Submitted filename: Response to Reviewers.docx [file pone.0329765.s001.docx]

**REVIEWER #1**

1- In Section 3.1.2, the authors use both Min-Max Scaling and Z-score normalization for feature distributions. It would be helpful if the authors could justify the choice of these methods and explain when each one is applied. Providing examples of different scenarios where these methods are preferred would help readers better understand the reasoning behind their use and improve the transparency of the methodology.

**Response**: Thank you for your insightful comment regarding the use of Min-Max Scaling and Z-score normalization in Section 3.1.2. We appreciate the opportunity to clarify our rationale for selecting these methods and to provide further justification for when each normalization technique is applied. To address this, we have expanded Section 3.1.2 to include the required details.

2- There appears to be some confusion regarding the use of Min-Max Scaling and Feature Scaling in the paper. The authors should clarify the differences between these methods and explain why both were applied. If these are indeed distinct steps, a clear justification for their combined use is needed to avoid potential conflicts in the methodology. The readers might find it difficult to determine whether Min-Max Scaling or neural networks and gradient-based methods were used, so clarifying this point would enhance the clarity of the work.

**Response**:

We thank the reviewer for pointing out the potential confusion regarding the use of Min-Max Scaling and Feature Scaling in the paper. We recognize the importance of distinguishing between these techniques and their specific roles in the preprocessing pipeline. Below, we clarify their differences, explain why both were applied, and provide a justification for their combined use:

In sum, Min-Max Scaling: This technique normalises feature values within a defined range (e.g., [0,1]). It ensures that features with varying units or magnitudes are brought to a standard scale, which is essential for maintaining consistency across the dataset. Min-max scaling is particularly beneficial for features with bounded ranges, such as CPU utilization or memory consumption, where preserving relative differences is crucial.

On the other hand, feature Scaling (Z-score Normalization): This refers to standardizing features to have a mean of 0 and a standard deviation of 1. Z-score normalization is especially effective for features with Gaussian distributions or when centring and scaling are needed to optimize the performance of neural networks and gradient-based models.

3- In Algorithm 1, there is no clearly defined stop criterion, which could potentially lead to unnecessarily long running times if the iteration value (T) is set too high. For example, setting T to a large value may cause the algorithm to run for many iterations, even though convergence might occur much earlier. A predefined evaluation metric or maximum number of iterations should be established to ensure that the algorithm converges efficiently to an optimal solution.

**Response**:

Thank you for your valuable feedback. We agree with your observation that the absence of a clearly defined stop criterion could lead to inefficient running times, especially when the iteration value (T) is set too high. To address this concern, we will incorporate a predefined evaluation metric, such as a convergence threshold or a maximum number of iterations, to ensure that the algorithm halts once an optimal or satisfactory solution is reached. Additionally, we will include a discussion of the trade-off between computational cost and solution accuracy, allowing users to adjust these parameters based on the specific requirements of their problem. This will help ensure both efficiency and the desired performance of the algorithm.

**Old algorithm**

**Modified one:**

4- In Section 3.3.1, the authors describe the CNN-LSTM architecture but do not specify important details such as the number of layers in both the CNN and LSTM networks, the number of neurons in each layer, or how these models are combined. Including such details is essential for ensuring that the methodology can be replicated. A diagram or figure that illustrates the architecture would be beneficial. Using tools like plot_model from tensorflow.keras.utils to visualize the model or providing a model summary would help improve transparency and enable other researchers to replicate the work.

**Response**:

Thank you for your constructive feedback regarding the need for more details about the CNN-LSTM architecture in Section 3.3.1. We agree that specifying critical architectural details and providing a visualization of the model would enhance the transparency and reproducibility of the methodology. (Refer to Figure 3).

5- The authors mention using transfer learning based on pre-trained weights, but do not specify which pre-trained model was used. It would be helpful to include this information so that readers can understand the specific model from which the weights were transferred. Additionally, further details on how transfer learning was implemented in the context of this work would add depth to the methodology section.

**Response:**

Thank you for your valuable comment regarding the use of transfer learning in our work. We acknowledge the importance of specifying the pre-trained model and providing additional details about how transfer learning was implemented to enhance the clarity and reproducibility of the methodology. To address this, we have made the required updates to the manuscript (Subsection 3.3.2).

6- The paper does not describe how the proposed model was fine-tuned after the initial training. It would be beneficial for the authors to elaborate on the fine-tuning process, including any adjustments made to hyperparameters, learning rates, or other aspects of the model to improve its performance.

**Response**:

Thank you for your insightful comment regarding the fine-tuning process of the proposed model. We agree that elaborating on the fine-tuning steps would strengthen the paper and provide valuable insights into how the model's performance was optimized. To address this, we have expanded the Methodology section to include a detailed explanation of the fine-tuning process.

7- The authors state that the CNN-LSTM architecture was implemented in Python using either TensorFlow or PyTorch. It is important to be more precise and state explicitly which framework was used for the implementation. This clarification will help avoid any ambiguity and ensure that the methodology is reproducible.

**Response**:

Thank you for pointing out the need for greater clarity regarding the implementation framework for the CNN-LSTM architecture. We completely agree that specifying the framework is crucial for ensuring the reproducibility of our methodology. To address this, we have updated the manuscript to explicitly state that the CNN-LSTM architecture was implemented in Python using TensorFlow.

8- The authors use two different datasets in their experiments: the CloudSim tool data and the Google Cluster Trace dataset. However, there is no mention of the performance of the CNN-LSTM model in terms of Mean Squared Error (MSE) or other relevant metrics. The authors should include results on MSE or similar performance measures to assess the model's predictive capability.

**Response**:

Thank you for your constructive feedback regarding the inclusion of Mean Squared Error (MSE) or similar performance metrics to assess the predictive capability of the CNN-LSTM model. We acknowledge that providing such metrics would offer valuable insights into the model's accuracy and reliability in predicting workload demand.

To address this, we have revised the manuscript to include an evaluation of the CNN-LSTM model using MSE, Mean Absolute Error (MAE), and Root Mean Squared Error (RMSE) as performance metrics. (Refer to Subsection 3.3.3 Model Evaluation, please).

**REVIEWER #2**

1- Problem Statement: The introduction needs to clearly state the gap in existing solutions and how SLADRO uniquely addresses this gap. While contributions are listed, their connection to specific limitations in current methods is not explicit.

**Response**:

Thank you for the valuable feedback. The introduction has been revised to explicitly state the gap in existing solutions and how SLADRO uniquely addresses these limitations. While the contributions of SLADRO were mentioned, I now ensure that each one is directly linked to a specific issue with current methods.

2- Figures and Visualizations: Several figures (e.g., throughput, makespan) are mentioned but not adequately explained in the provided text. Ensure all figures are included and provide sufficient discussion on their relevance to the findings.

**Response**:

Thank you for highlighting the importance of ensuring that all figures in the manuscript are adequately explained and discussed. We agree that figures such as those illustrating throughput, makespan, resource utilization, and energy efficiency are critical to conveying the relevance and significance of our findings.

3- The figure captions (e.g., Fig 4, Fig 5) should be more descriptive to effectively convey the role of each figure without relying solely on the text. Consider adding details about what specific scenario or comparison is illustrated in each figure.

**Response**:

Thank you for your valuable feedback regarding the need for more descriptive figure captions. We agree that clearer captions can enhance the reader's understanding of the figures without relying solely on the accompanying text. To address this, we have revised the captions for the figures in the manuscript to include more detailed descriptions.

4- Comparative Analysis: The manuscript claims SLADRO outperforms other methods, but the benchmarks (e.g., Round Robin, Least Connections) are relatively basic. Include comparisons with more advanced techniques like hybrid metaheuristics or AI-driven approaches (e.g., DPSO-GA, HBA-Z).

**Response**:

Thank you for your valuable feedback on the comparative analysis. We appreciate the suggestion to include comparisons with advanced techniques such as hybrid metaheuristics or AI-driven approaches (e.g., DPSO-GA, HBA-Z). We would like to clarify that the manuscript already includes comparisons with several advanced and recent mechanisms, as referenced in the Results and Discussion section. These mechanisms include:

1. LBMPSO: (Reference 18)
2. HHO-PIO: (Reference 19).
3. QMPSO: (Reference 20).
5- Scalability Testing: There is limited discussion on how SLADRO performs under varying workloads or in larger-scale cloud environments. Address scalability and robustness in more detail.

**Response**:

Thank you for your valuable feedback regarding the limited discussion on scalability and robustness of SLADRO. We agree that scalability is a critical factor in evaluating the effectiveness of load-balancing algorithms in cloud environments. To address this, we have expanded the discussion on SLADRO’s performance under varying workloads and larger-scale cloud environments in the manuscript.

6- DRL Implementation: The description of the DQN-based scheduling approach is detailed but lacks clarity on hyperparameter settings and the exploration-exploitation balance (e.g., epsilon decay in the epsilon-greedy policy).

**Response**:

We sincerely thank the reviewer for their valuable feedback regarding the description of the DQN-based scheduling approach, particularly the need for clarity on hyperparameter settings and the exploration-exploitation balance. In response to this comment, we have added a detailed table (Table 1) to the manuscript under the Methodology section in the Load Balancing Strategy Implementation subsection

7- Feature Selection: While OOA-PSO is presented as a novel approach, the paper does not sufficiently justify why it is superior to other optimization techniques for feature selection.

**Response**:

We sincerely thank you for highlighting the need to better justify the superiority of the OOA-PSO approach for feature selection. In response to this comment, we have added a detailed comparison and justification in the Methodology section under the Feature Selection subsection.

8- The manuscript contains grammatical errors and awkward phrasing. For instance, "Fig 3 focuses on throughput..." and "Moving to 5, which shows the makespan..." lack professional tone and clarity. A thorough language edit is necessary.

**Response**:

We sincerely thank the reviewer for pointing out the need for grammatical improvements and adjustments to the tone and phrasing in the manuscript. In response, we have conducted a thorough language edit throughout the manuscript to address grammatical errors and enhance clarity, professionalism, and readability.

9- Although energy consumption is highlighted as a key metric, there is insufficient explanation of how SLADRO achieves lower energy usage compared to baseline methods. Expand on the mechanisms that contribute to energy efficiency.

**Response**:

Thank you for your insightful comment regarding the need for a detailed explanation of how SLADRO achieves lower energy usage compared to baseline methods. We acknowledge that energy efficiency is a key metric in cloud computing and are pleased to expand on the mechanisms underlying SLADRO’s energy-saving capabilities
