# Supplementary material for: Smart load balancing in cloud computing: Integrating feature selection with advanced deep learning models
Source: PLoS One. 2025 Sep 9;20(9):e0329765. doi: 10.1371/journal.pone.0329765 (PMC12419607; doi:10.1371/journal.pone.0329765)
Supplement: Attachment — Submitted filename: Response to Reviewers_ Round 2.docx [file pone.0329765.s002.docx]

**REVIEWER #2**

**Comment No 1:** Comparative Analysis: More discussion is needed on the limitations of SLADRO compared to other state-of-the-art hybrid models, especially regarding computational cost and scalability.

- **Response:**

We appreciate the reviewer’s suggestion to further discuss the limitations of SLADRO compared to state-of-the-art hybrid models, particularly in terms of computational cost and scalability. In response, we have expanded the discussion in the Limitations and Future Work. The revised manuscript now details SLADRO’s computational overhead due to deep learning and reinforcement learning integration, its scalability challenges in handling large-scale cloud workloads, and comparisons with alternative hybrid models, including GA, PSO, and advanced RL-based approaches. Additionally, we propose future optimizations such as model pruning, distributed processing, and adaptive reinforcement learning to enhance efficiency. We thank the reviewer for their insightful feedback, which has strengthened the comparative analysis of SLADRO.

**Comment No 2:** SLADRO is now compared against DPSO-GA and HBA-Z, but the discussion remains limited. The authors should provide statistical significance testing (e.g., t-tests) to validate SLADRO’s superiority over DPSO-GA and HBA-Z methods.

- **Response:**

We thank the reviewer for their insightful comment. In response, we have incorporated statistical significance testing, including t-tests, to rigorously validate SLADRO's superiority over the SOTA methods. These tests have been included in the manuscript, comparing SLADRO against SOTA across all performance metrics, such as throughput, makespan, processing power, resource utilization, and energy consumption. The results, presented in the updated manuscript, demonstrate that SLADRO significantly outperforms both methods, with all p-values indicating strong statistical significance. This addition enhances the robustness of our evaluation and provides a comprehensive comparison as requested.

**Comment No 3:** Scalability Testing: The manuscript now mentions large-scale workloads, but no experiments are provided on how SLADRO performs under extreme workloads. The authors should include tests on larger-scale datasets (e.g., AWS workload traces).

- **Response:**

We appreciate the reviewer’s insightful comment. To address this concern, we have added Section 5.2 - Large-Scale Workloads Testing to the manuscript. This section evaluates the scalability of the proposed SLADRO model using the Google 2019 Cluster Sample dataset, which provides workload traces from Google’s cloud infrastructure. The experiments demonstrate SLADRO’s ability to efficiently handle large-scale workloads, showcasing its robustness under extreme conditions.

Additionally, we acknowledge the importance of further evaluations on diverse datasets. As part of future work, we plan to extend our experiments by incorporating additional large-scale datasets, such as AWS workload traces, and benchmarking SLADRO against more recent load-balancing models.

**Comment No 4:** Justification for OOA-PSO/Features Selection: The authors compare OOA-PSO with GA, PSO, and ACO, showing superior feature selection accuracy. The authors should explain why OOA-PSO works better for this specific problem beyond raw performance.

- **Response:**

We appreciate the reviewer’s insightful comment regarding the justification of OOA-PSO’s superiority beyond performance metrics. To address this, we have added a detailed explanation in the revised manuscript.

The enhanced performance of OOA-PSO in feature selection is attributed to its ability to balance exploration and exploitation efficiently. The OA component ensures a systematic and diverse exploration of the feature space, preventing premature convergence and avoiding local optima—an issue commonly observed in GA, PSO, and ACO. Unlike GA, which depends on crossover and mutation operations that may introduce randomness and inconsistency in selected features, OOA-PSO ensures stable and optimal feature selection. Additionally, PSO alone may converge too quickly to suboptimal solutions, whereas the structured sampling of OA enhances feature diversity, leading to improved generalization.

For cloud load balancing, where workload patterns fluctuate dynamically, rapid and accurate feature selection is crucial. OOA-PSO’s structured approach efficiently reduces dataset dimensionality, lowering computational overhead while preserving high predictive accuracy. Our experimental results further validate this, demonstrating that OOA-PSO achieves superior feature selection accuracy (95.2%) with fewer convergence iterations compared to GA, PSO, and ACO. These improvements make OOA-PSO particularly well-suited for real-time resource allocation in cloud environments.

This justification has been incorporated into the revised manuscript in Section 3.2.2 (Feature Selection Algorithm: OOA-PSO). We thank the reviewer for their valuable feedback, which has helped improve the clarity and depth of our explanation.

**Comment No 5:** Energy Efficiency Explanation: The authors mention that SLADRO improves energy efficiency but do not explain the mechanism in detail. They should discuss how DRL and feature selection contribute to energy efficiency (e.g., fewer VM migrations, lower idle CPU usage).

- **Response:**

We appreciate the reviewer’s insightful comment. To clarify, SLADRO improves energy efficiency through DRL-based intelligent scheduling, which minimizes VM migrations and idle CPU usage, and OOA-PSO feature selection, which reduces computational overhead. We have added a detailed explanation in Section [5.3] of the revised manuscript to highlight these mechanisms and their impact on energy efficiency.

**Comment No 6:** Reproducibility: While the implementation details are robust, additional clarity on hyperparameter tuning, training duration, and computational resources used would improve reproducibility.

- **Response:**

We appreciate the reviewer’s valuable feedback on improving reproducibility. To address this, we have provided detailed information on hyperparameter tuning, training duration in Appendix I. This section includes comprehensive tables outlining the optimized hyperparameters for CNN-LSTM and DQN models, the hardware and software configurations used, and the total training time required for both models. These additions ensure greater clarity and facilitate the replication of our methodology. We thank the reviewer for their insightful suggestion, which has helped improve the completeness of our implementation details.

Please note that the computational resources used to conduct the expirments are already discussed in subsection 4.4.

**Comment No7:** Writing & Formatting Issues: There are minor typographical errors and awkward phrasing in some sections (e.g., "Litarture review" should be "Literature Review").

- **Response:**

We appreciate the reviewer’s feedback on writing and formatting issues. We have carefully reviewed the manuscript and corrected typographical errors, including changing "Litarture review" to "Literature Review." Additionally, we have refined awkward phrasing in relevant sections to improve clarity and readability.

**Comment No 8:** Justify the choice of ϵ and Tmax in Algorithm 1 (stop criterion).

- **Response:**

We appreciate the reviewer’s comment. We have added a detailed justification for the choice of ϵ (convergence threshold) and Tmax (maximum iterations) in Algorithm 1. The revision explains how ϵ ensures a balance between optimization accuracy and computational efficiency, while Tmax prevents excessive computation and ensures thorough exploration of the search space. These additions are now included in the methodology section to clarify the stopping criteria.

**Comment No 9:** Explain why transfer learning is necessary in this problem.

- **Response:**

We appreciate the reviewer’s insightful comment. Transfer learning is necessary in this problem to leverage pre-trained models, such as ResNet50, for feature extraction, reducing the need for extensive labeled training data and computational resources. Since cloud workload prediction involves analyzing complex spatial and temporal dependencies, transfer learning allows the CNN component to extract meaningful workload-related features efficiently. By using pre-trained weights, the model benefits from hierarchical feature representations, leading to faster convergence, improved accuracy, and better generalization to varying workload patterns. This integration significantly enhances the model’s performance compared to training from scratch. The methodology section is revised accordingly.

**Comment No 10:** Add statistical significance testing for performance comparisons.

- **Response:**

We thank the reviewer for their insightful comment. In response, we have incorporated statistical significance testing, including t-tests, to rigorously validate SLADRO's superiority over the SOTA methods.

**Comment No 11:** Provide additional scalability experiments for large cloud workloads.

- **Response:**

We appreciate the reviewer’s insightful comment. To address this concern, we have added Section 5.2 - Large-Scale Workloads Testing to the manuscript. This section evaluates the scalability of the proposed SLADRO model using the Google 2019 Cluster Sample dataset, which provides workload traces from Google’s cloud infrastructure. The experiments demonstrate SLADRO’s ability to efficiently handle large-scale workloads, showcasing its robustness under extreme conditions.

Additionally, we acknowledge the importance of further evaluations on diverse datasets. As part of future work, we plan to extend our experiments by incorporating additional large-scale datasets, such as AWS workload traces, and benchmarking SLADRO against more recent load-balancing models.

**Comment No 12:** Expand the discussion on energy efficiency mechanisms.

- **Response:**

We appreciate the reviewer’s insightful comment. To clarify, SLADRO improves energy efficiency through DRL-based intelligent scheduling, which minimizes VM migrations and idle CPU usage, and OOA-PSO feature selection, which reduces computational overhead. We have added a detailed explanation in Section [5.3] of the revised manuscript to highlight these mechanisms and their impact on energy efficiency.
